# Performance profiling of the unit trust funds in Malaysia with data mining techniques

**DOI:** 10.12688/f1000research.73467.1

**Published:** 2021-12-13

**Authors:** Aida Farah Khairuddin, Keng-Hoong Ng, Kok-Chin Khor

**Affiliations:** 1 Faculty of Computing and Informatics, Multimedia University, Cyberjaya, 63000, Malaysia; 2 Lee Kong Chian Faculty of Engineering and Science, Universiti Tunku Abdul Rahman, Sungai Long, 43200, Malaysia

**Keywords:** unit trust funds, expectation maximisation, apriori, performance profiling

## Abstract

Background: Millennials are exposed to many investment opportunities, and they have shown their interest in gaining more income via investments. One popular investment avenue is unit trusts. However, analysing unit trusts’ financial data and gaining valuable insights may not be as simple because not everyone has the required financial knowledge and adequate time to perform in-depth analytics on the numerous financial data. Furthermore, it is not easy to compile the performance of each unit trust available in Malaysia. The primary objective of this research is to identify unit trust funds that provide higher returns than their average peers via performance profiling.

Methods: This research proposed a performance profiling on Malaysia unit trust funds using the two data mining techniques, i.e., Expectation Maximisation (EM) and Apriori, to assist amateur retail investors to choose the right unit trust based on their risk tolerance. EM clustered the unit trust funds in Malaysia into several groups based on their annual financial performances. This was then followed by finding the rules associated with each cluster by applying Apriori. The resulted rules shall serve the purpose of profiling the clustered unit trust funds. Retail investors can then select their preferred unit trust funds based on the performance profile of the clusters.

Results: The yearly average total return of the financial year 2018 and 2019 was used to evaluate unit trust funds’ performance in the clusters. The evaluation results indicated that the profiling could provide valuable and insightful information to retail investors with varying risk appetites.

Conclusions: This research has demonstrated that the financial performance profiling of unit trust funds could be acquired via data mining approaches. This valuable information is crucial to unit trust investors for selecting suitable funds in investment.

## Introduction

In Malaysia, different investment securities or schemes are publicly available to investors. The common securities include stocks, bonds, deposits, properties, unit trusts and commodities. Youngsters nowadays are more aware of the importance of earning passive income from investment. Bursa Malaysia recorded a 36% rise in the new Central Depository System (CDS) account holders aged below 25 in 2016, totalling more than 25.2 thousand account holders at the end of this year. ^1^ Recently, the fast-growing trend has driven more financial products to be brought to the market to attract investors.

Unit trusts are investment options with lower risk ^2^ than equities because they are well-diversified financial instruments that fund managers handle. Each unit trust fund has its investment objective and strategy. Hence an amateur needs to pick the suitable funds to be included in their portfolio. However, it is not easy to choose suitable unit trust funds that meet the investor’s requirements, especially from a large pool of unit trust funds available in Malaysia. As of December 2019, the Security Commission of Malaysia ( www.sc.com.my/analytics/fund-management-products) reported that Malaysia’s total launched unit trust funds are 685.

Amateur investors always find it challenging to identify appropriate unit trust funds that meet their investment strategies and risks. The task requires much time and effort to complete, looking at the abundance of data to be searched and analysed. Most amateur investors are usually busy with their full-time jobs or running their own business. As a result, they cannot screen and perform in-depth analysis on many unit trust funds.

Another typically encountered problem is the low financial literacy among amateur investors. Thus, they cannot carry out a thorough analysis to extract meaningful information or knowledge from the annual reports they read. Subsequently, they find it challenging to analyse unit trust funds’ performance, and usually, their findings are inconclusive and indecisive. Amateur investors also face obstacles when they are looking for unit trust funds to fulfil their risk appetite. Hence, it is difficult for them to make investment decisions.

Considering the mentioned problems, this article aims to propose a data mining model that could build the performance profiles of unit trust funds using clustering and association rules mining algorithms. The model could mitigate the problems because: (i) the performance profiles could be generated rapidly, (ii) the financial information in the profile is intuitive and easy to understand by amateur investors, and (iii) the profiles also provide risk information.

## Literature review

The research by F. Cai *et al*. ^3^ evaluated 904 investment funds from financial datasets (time series and transactions) retrieved from the Morningstar using *k*-means and density-based clustering. The finding indicated that *k*-means performed better than the latter, giving the best number of clusters. In the research by T. Sakakibara *et al.,* ^4^ clustering of mutual funds was done based on investment similarity instead of using historical performance similarity. The proposed approach was tested on 551 Japanese mutual funds. The result claimed that the approach could acquire the optimal number of clusters, even better than the classification provided by Morningstar Inc.

K. H. Ng *et al.* ^5^ adopted association rules mining to find frequent financial patterns in outlier stocks listed on Bursa Malaysia. The outliers were identified via a score-based approach, and they were manually grouped into superior and poor outliers. The research produced nine rules associated with outstanding stocks and four rules for the poor performing stocks. Investors can refer to these rules for decision making. Another study applied clustering to construct an efficient stock portfolio in the Warsaw Stock Exchange. ^6^ The study utilised *k*-means and Partitioning Around Medoids (PAM) methods for this purpose and showed satisfactory returns.

## Methods

This research applies data mining techniques to profile the unit trust funds available in Malaysia. The entire process consisted of five key steps. The first step was to collect the financial data of the unit trust funds from annual reports. The next step involved a normalisation technique to ensure that the financial attributes share the same scale. The third step was grouping unit trust funds using a clustering algorithm. This was followed by profiling the clusters to find the common rules/characteristics associated with each cluster. In the final step, evaluation was conducted to study the efficiency of the proposed profiling method. The research overview is shown in Pseudocode 1.
**Pseudocode 1. Profiling of unit trust funds.**
**Input:** Annual financial reports of unit trust funds, *AFRs*
**Output:** Financial profiling of unit trust funds

1. //collect unit trust funds financial data
2. for each unit trust fund’s report, *ut* ϵ *AFRs* do
3.  for *Fy* ϵ {*fromYr*, *toYr*} do//financial year *Fy*
4.   *FinD* ← extract *Fy* data from *ut*
5.  //append financial data, *FinD* to dataset *Db_Fy_*
6.   *Db_Fy_.* append (*FinD*)
7.  end for
8. end for
9. //normalize the two financial datasets
10. perform z-score normalization on *Db_2017_* and *Db_2018_*
11. //apply EM clustering to group unit trust funds
12. {*C_1_*, *C_2_*, *C_3_*, … , *C_m_*} ← EMCluster (*Db_2017_*)
13. //EM generated *m* clusters, *C_1_* until *C_m_*
14. {*E_1_*, *E_2_*, *E_3_*, … , *E_n_*} ← EMCluster (*Db_2018_*)
15. //EM generated *n* clusters, *E_1_* until *E_n_*
16. //discretize the attributes in the datasets
17. calculate quartile 1 – 3 values for all attributes in *Db_2017_* and *Db_2018_*
18. for each attribute, *att* ϵ {*Db_2017_*, *Db_2018_*} do
19.   if *att.value* >= q3//quartile 3 value
20.    a*tt.value* ← high
21.   else if *att.value* > = q1
22.    a*tt.value* ← moderate
23.   else
24.    a*tt.value* ← low
25.   end if
16. end for
27. //find associated rules in each cluster
28. for each cluster generated in line 12 and 14
29.   apply apriori algorithm to find frequent itemset for the cluster
30.   profile the cluster based on the frequent itemset
31. end for


### Unit trust funds datasets

The financial data of the unit trust funds were gathered from the annual report of each unit trust fund. From the annual report, six common financial variables were identified as crucial in the study: Net Asset Value (NAV) per unit, total growth rate, capital growth rate, income distribution rate, management expense ratio (MER), and portfolio turnover ratio (PTR). The detail of each financial attribute is shown in Table 1. In this study, 326 local unit trust funds’ data from 26 financial institutions were collected (Underlying data). ^7^ Examples of financial institutions were CIMB Bank, Maybank, Prudential Insurance, Kenanga Investment Bank, etc.

**Table 1.** The six selected financial attributes that were used in this study.

Financial attribute	Description/Formula
NAV per unit (RM)	The net asset value of a fund divided by the number of units in circulation at the valuation point. *NAV per unit (RM)* = *total NAV of fund*/ *total units*
Total Growth (%)	The actual rate of return of an investment at the valuation point. *Total Growth = Capital Growth (%)* + *Income Return (%)*
Capital Growth (%)	Increase in the value of an asset over time. *Capital Growth = (NAV per unit end*/ *NAV per unit begin* – *1) x 100*
Income Distribution (%)	Income declared and distributed back to its investors in a year *Income Distribution = (Gross Income Distribution declared/NAV per unit begin - 1) x 100*
Management Expense Ratio (MER) (%)	A measure to see how expensive a unit trust fund is to investors. ^7^ *MER = (operating costs + management fee) /Total Assets under management*
Portfolio Turnover (PTR) (times)	A measure of how frequently assets within a fund are bought and sold by the managers. ^7^ *X = total purchases or total sales (choose higher)* *PTR = X/average monthly assets*

### Data normalisation

The created dataset underwent the z-score normalisation before the clustering process. This was to prevent features with a larger scale from outweighing smaller-scaled features in the data mining process. This normalisation technique produced equal weight features within the range of [−1,1]. The formula for the z-score normalisation ^8^ is shown in the following:

$$Z=\left(p-\mu \right)/\sigma$$

Variable *p* denotes the original financial data value. The mean and the standard deviation of the financial data are represented by *μ* and *σ,* respectively.

### Expectation Maximisation (EM) clustering

In this step, the normalised dataset was partitioned into clusters based on their feature similarity. The use of EM clustering in this study could be justified with the following: (i) No predefined number of clusters, and (ii) It could handle missing values. ^9^ EM clustering assigns each unit trust fund a probability distribution that represents the probability it belongs to each cluster. By maximising the log-likelihood of the data, EM finds the optimised parameters of a probability distribution.

EM has two major steps, i.e., E-step (expectation) and M-step (maximisation). The detailed explanation of the algorithm is illustrated in Pseudocode 2. EM begins with initialising random values to parameters mean, variance, and a fraction of the data in each cluster *c* (line 2). Subsequently, E-step (line 3-7) computes the expected likelihood for the unit trust dataset. This is followed by maximising the likelihood of the data by re-estimating the parameter values in the M-step (lines 8-10). Both steps are repeated until the likelihood converges and reaches a local maximum. This means that the iteration will halt once the likelihood cannot be improved further.
**Pseudocode 2. EM clustering on the unit trust dataset.**
**Input:** Unit trust dataset for the financial year, *Db_FY_*
**Output:** Unit trust clusters

1. //*Fr_c_* represents the fraction of the data represented by cluster *c*
2. assign random values to *mean^0^*(*c*), *variance^0^*(*c*) and *Fr^0^*_c_
3. for each unit trust, *utr* in *Db_yr_* do //expectation step
4.  for each cluster *c* do
5.   compute the probability of *utr* in the cluster *c*
6.  end for
7. end for
8. for each cluster *c* do//maximisation step
9.  re-estimate *mean^i+1^*(*c*), *variance^i+1^*(*c*) and *Fr^i+1^*_c_ to maximise the likelihood of the unit trusts
10. end for
11. repeat steps 3 – 10 until the parameters converge
12. return the final clusters, *c_1_*, *c_2_* … , *c_k_* //*k* denotes the number of clusters


### Class Association Rules (CAR) mining

The original format of the financial features in the dataset is numeric. Thus, these data were required to undergo discretisation before being processed by the association rules algorithm. The binning method was applied to discretise the financial features into three categories, i.e., low, medium and high. They were divided using the quartile range. The data was discretised to “low” if it was less than Q1 of the data (quartile 1). “Moderate” referred to data that was more than or equal to Q1 and less than or equal to Q3. Any data greater than Q3 was discretised as “high”.

Association Rules Mining (ARM) is useful to discover frequent rules or patterns among groups of objects in a dataset. In this study, CAR ^10^ mining was adopted to find a subset of rules associated with each cluster. The primary intention was to uncover a set of financial behaviours that are associated with each cluster uniquely. The financial behaviours can be used to build the cluster profile. CAR mining could be divided into two major steps, i.e. (i) discover all frequent *k*-itemsets in a dataset that comply with the user-defined minimum support, (ii) find the frequent *k* + 1 itemsets with the help of *k*-itemsets by applying a self-join rule. The detailed steps of the CAR are described in Pseudocode 3.
**Pseudocode 3. Mining rules associated with the cluster.**
**Input:** Discretised financial data of unit trusts in a cluster
**Output:** Financial profiles of the unit trust cluster

1. //*mSup* denotes minimum support
2*. L_1_* ← search all frequent 1-itemset with support count >= *c_size* * *mSup* //*c_size* is the cluster size
3. Initialize *j* ← 1
4. repeat
5.  *j* ← *j*+1
6.  *CandIS_j_* ← find candidate itemsets from *L_j-1_*
7.  for each unit trust *utc* in the cluster do
8.   *Cand_utc_* ← subset (*CandIS_j_*, *utc*)
9.   for each candidate itemset in *Cand_utc_* do
10.    add support count of the candidate itemset
11.   end for
12.  end for
13.  *L_j_* ← extract the frequent *j*-itemsets with support count >= *c_size* * *mSup*
14. until *L_j_* is null
15. return all *L*


## Results and discussion

This study used a unit trust dataset that contained six normalised financial variables of the year 2017. It started with the clustering process using the Expectation Maximisation (EM) method. As a result, eight clusters were produced. Table 2 shows the detail of each cluster, including the number of assigned unit trust funds and the means of the six financial features. Cluster 2 was the largest cluster with 63 unit trust funds, and the smallest was Cluster 5 with only 14 members.

**Table 2.** EM clustering yielded 8 clusters. The smallest cluster has 14 funds (cluster 5), and the largest cluster contains 63 funds (cluster 2).

NAV	TR	CG	ID	MER	PTR
**Cluster 1** (42 unit trust funds)					
‒0.177	‒0.033	‒0.318	‒0.356	‒0.178	‒0.339
**Cluster 2** (63 unit trust funds)					
‒0.239	‒0.102	‒0.208	‒0.967	‒0.139	‒0.513
**Cluster 3** (52 unit trust funds)					
‒0.572	‒0.027	‒0.364	‒0.264	‒0.039	‒0.454
**Cluster 4** (41 unit trust funds)					
‒0.444	‒0.196	‒0.429	‒0.622	‒0.399	‒0.727
**Cluster 5** (14 unit trust funds)					
‒0.426	‒0.124	‒0.487	0.055	0.002	‒0.375
**Cluster 6** (38 unit trust funds)					
‒0.776	‒0.096	‒0.239	‒0.870	‒0.128	‒0.850
**Cluster 7** (49 unit trust funds)					
0.207	‒0.207	‒0.449	‒0.600	‒0.588	‒0.754
**Cluster 8** (27 unit trust funds)					
‒0.133	**0.514**	**0.409**	‒0.810	‒0.003	‒0.233

Even though the mean of each financial variable is produced and displayed in the table, it is not an easy task for an amateur investor to analyse and interpret the generated information correctly. This is because the financial variables have been normalised before the clustering. Hence, a further step was taken to process this information by employing the Association Rules Mining technique so that the generated profile information on each cluster would be more intuitive and easier to understand. To perform the ARM, the continuous financial data was discretised into three bins, i.e., high, moderate, low, using the binning method.

ARM was conducted on each cluster to discover a set of rules associated with each cluster. Two parameters, i.e., minimum support and minimum confidence, were predefined. The first parameter was set to 50%, ^11^ and a higher threshold of 90% was applied to the minimum confidence. ARM only yielded meaningful outcomes on three clusters: Cluster 8, Cluster 4, and Cluster 5. The remaining clusters did not demonstrate any strongly associated rules after the process.

Cluster 8 produced two frequent 3-itemsets ( Table 3). The result strongly indicates that unit trust funds in this cluster belong to the type of high total return, high capital growth, and high portfolio turnover. Upon examining the portfolios of some unit trust funds in the cluster, it was discovered that these funds prioritise stock investment in their investment baskets. This finding was sufficient to justify the derived rules associated with the cluster because the return and capital growth of stock investment were relatively higher than the fixed income securities such as bonds, fixed deposits, etc. High portfolio turnover is also expected in these unit trust funds because the fund managers must always fine-tune their portfolios to maximise the returns. As such, the financial profile of this cluster can be summarised as “Aggressive”. This cluster is appropriate for investors who are risk-takers and aim for a high growth rate and return.

**Table 3.** ARM on the discretised financial data of the unit trust funds in Cluster 8.

Itemsets	Support count
Total Return = **High** Capital Growth = **High** Income Distribution = Moderate	23
Total Return = **High** Capital Growth = **High** Portfolio Turnover = **High**	18

The rules associated with Cluster 4 are illustrated in Table 4, displaying the three frequent 3-itemsets derived from the cluster. The financial profile of Cluster 4 might not have been as outstanding as Cluster 8, but it is noteworthy to describe the unit trust funds in Cluster 4 as average and Defensive. Even though they have average performance among their peers in total return, capital growth, and income distribution, they give stable returns over the years.

**Table 4.** Association rules mining applied to Cluster 4.

Itemsets	Support count
NAV per unit = Moderate Capital Growth = Moderate Income Distribution = Moderate	24
NAV per unit = Moderate Total Return = Moderate Income Distribution = Moderate	23
NAV per unit = Moderate Total Return = Moderate Capital Growth = Moderate	21

A thorough analysis of some of the unit trust funds in Cluster 4 revealed conservative investment approaches. They adopted a more diverse investment strategy by pooling their funds into a basket of securities consisting of shares and bonds, deposits, and properties. Combining these investment securities can lower the downside risk due to market volatility. In short, Cluster 4 was considered “Defensive”, and therefore suitable for investors with a low-risk tolerance.

Cluster 5 exhibited inferior financial performance. Table 5 shows the six frequent 2-itemsets derived after the ARM process. The associated rules in this cluster included low total return, low capital growth, high portfolio turnover, and high-income distribution. The phenomena of low total return but high-income distribution was due to poor capital growth. Stagnant or negative growth in the investment capital significantly outweighed the income distribution. Hence, this cluster was deemed as an inferior type. Investors are advised to avoid such unit trust funds at all costs.

**Table 5.** Six frequent 2-itemsets were produced after analysing Cluster 5 with the ARM.

Itemsets	Support count
NAV per unit = Moderate Income Distribution = High	10
Total Return = **Low** Capital Growth = **Low**	8
NAV per unit = Moderate Total Return = **Low**	7
Total Return = **Low** Income Distribution = High	7
NAV per unit = Moderate Portfolio Turnover = High	7
Income Distribution = High Portfolio Turnover = High	7

**Figure 1.**
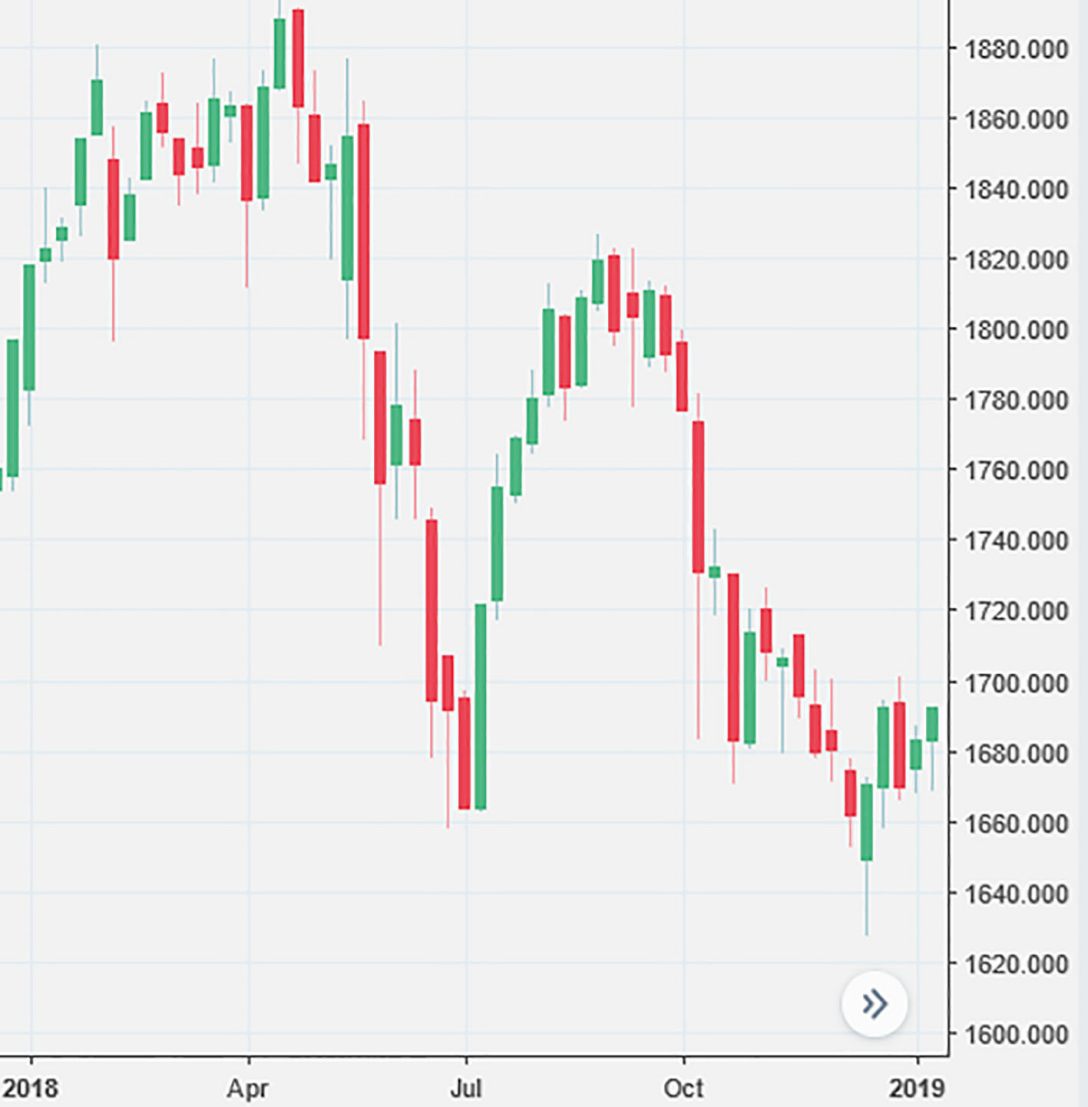
Bursa Malaysia KLCI Index lost more than 100 points in the year 2018.

The financial profiles created for the three clusters were further assessed and validated using the average total return (yearly) for the financial year 2018 and 2019 ( Table 6). For 2018, the best performer was the Defensive cluster, which was the only one that still delivered a positive return (+1.17%). Both Aggressive and Inferior clusters were in the negative territory. Thorough investigation revealed that the unsatisfactory performance in 2018 was mainly attributed to the tumble of the Bursa market ( Figure 1) affected by the three factors: general election, funds pulled out by foreign investors, and negative investor sentiment.

**Table 6.** Three identified clusters were evaluated using average total returns (%) in 2018 and 2019.

Cluster	Average total return (%)		
	2017	2018	2019
Cluster 8 **Aggressive**	+21.56	‒9.41	+3.52
Cluster 4 **Defensive**	+3.07	**+1.17**	**+4.65**
Cluster 5 **Inferior**	+3.94	‒8.69	‒2.98

The 14th Malaysia general election has shaken investors’ confidence to invest in Malaysia’s equity markets. As a result, many investors withdrew their securities investments, and their action caused a sharp fall in the Bursa Index. ^12^ The stock market was still in higher volatility mode in the second half of 2018. Many stocks could not recover their prices in the first half of the year. ^13^ Funds pulled out by foreign fund managers also attributed to the stock market fall. It has been reported that the net foreign fund outflow in 2018 for Malaysia totalled RM11.65 billion. ^14^ The last factor that adversely impacted the stock market was investor sentiment. Issues such as unresolved trade wars, lower oil prices, and geopolitical tension had refrained investors from securities investment.

In 2019, the volatility of the Malaysian stock market had subsided. Hence, all three clusters showed improvement in the total return as compared to the preceding year. The average total return (+4.65%) of Cluster 4 outperformed the others. Cluster 8 managed to gain a positive return of +3.52%. The worst performer (−2.98%) was still Cluster 5. The evaluation of two financial years for the three cluster profiles has strongly demonstrated that Cluster 4, with a Defensive profile, was resilient in the harsh economic climate that included market downturn, high market volatility, etc. This cluster may not have performed as well as Cluster 8 with an Aggressive profile during the economic boom, but it did provide steady passive income with lesser risk in the long run. Lastly, it could be summarised that investors with lower risk tolerance could aim at the defensive unit trust funds in Cluster 4. On the other hand, high-risk investors could identify the Aggressive unit trust funds in Cluster 8.

## Conclusions

This study used data mining techniques to generate a financial profile for unit trust funds. The profile information is intuitive and easily understood by an investor. The investor could narrow down the number of unit trust funds for analysis. Investors could focus on just a single cluster for making an investment decision. For instance, the investor could identify aggressive unit trust funds from Cluster 8 if he/she is a high risk-taker.

On the contrary, investors could use Cluster 4 for steady and average passive incomes. The finding from this study will have significant implications for unit trust investors. To the best of our knowledge, no published work has applied data mining approaches to profile unit trust funds in Malaysia. We hope to continue and extend the study to include non-financial factors, e.g., fund manager profile and time-series data, in the future.

## Data availability

### Underlying data

Zenodo: Malaysia unit trust funds dataset, https://doi.org/10.5281/zenodo.5291931. ^7^

Local unit trust checked version 1: The dataset contained 326 unit trust funds with their six financial attributes.

Data are available under the terms of the Creative Commons Zero “No rights reserved” data waiver (Attribution 4.0 International)

## Author contributions

All authors contributed equally to the conceptualisation of this study. Data curation, methodology, analysis and investigation, were performed by Aida Farah Khairudin. Keng-Hoong Ng and Kok-Chin Khor supervised the study and validated the evaluation results. Keng-Hoong Ng and Aida Farah Khairudin wrote the first draft of the manuscript. Kok-Chin Khor reviewed and edited the manuscript. All authors checked and approved the final manuscript.
