# Supplementary material for: Performance profiling of the unit trust funds in Malaysia with data mining techniques
Source: F1000Res. 2021 Dec 13;10:1272. [Version 1] doi: 10.12688/f1000research.73467.1 (PMC9237553; doi:10.12688/f1000research.73467.1)
Supplement: Peer review history

# Peer review: Reviewer response for version 1

**DOI:** 10.5256/f1000research.77120.r109294

**Published:** 2022-06-27

**Authors:** Wen Cheong Chin

**Affiliations:** 1 Department of Mathematics, Xiamen University Malaysia, Sepang, Malaysia

This article aims to use 2 data mining methodologies to profile the performance of 326 unit trust funds in Malaysia. 6 financial attributes have been selected and has being normalized as an Z-score before the clustering processes. The outcome of the analysis is able to assist investors in their investments based on their risk and return preferences by selecting their desired Cluster. 

 Suggestion:

 1. Provide more literature review related to this similar study in profiling performance.

 2. It is good to provide some risk indicator (eg. Sharpe ratio) for each cluster. Therefore investors can consider how risky each cluster.

 3. It is good for this study to extend to predictive analysis, where investors are given some guides for their future investments.

Is the work clearly and accurately presented and does it cite the current literature?

Partly

If applicable, is the statistical analysis and its interpretation appropriate?

Yes

Are all the source data underlying the results available to ensure full reproducibility?

Yes

Is the study design appropriate and is the work technically sound?

Yes

Are the conclusions drawn adequately supported by the results?

Yes

Are sufficient details of methods and analysis provided to allow replication by others?

Yes

Reviewer Expertise:

Financial time series and risk management.

I confirm that I have read this submission and believe that I have an appropriate level of expertise to confirm that it is of an acceptable scientific standard.

# Peer review: Reviewer response for version 1

**DOI:** 10.5256/f1000research.77120.r109286

**Published:** 2022-02-23

**Authors:** Lay Ki Soon

**Affiliations:** 1 School of Information Technology, Monash University Malaysia, Subang Jaya, Malaysia

The article reports the experiments on profiling selected unit trust funds in Malaysia using EM and CAR algorithms. While the results were as expected, the overall work is decent and the experimental validation is quite thorough. Some minor suggestions to improve the article:

 1. More details should be provided on the dataset collection. For instance, the source for the raw data (annual report), the year of the annual report collected, and how were the 326 funds selected.

 2. In terms of the features, why were only six variables used? Any empirical results to support this?

 3. For the discretization, which binning method was used?

 4. In results analysis, details were given on the impact of general elections to the stock market in Malaysia. It would be good to include relation between the stock market and unit trust funds.

 5. The results were good in the sense that similar unit trust funds are grouped in the same clusters. These were expected. Any outliers (something that is unexpected but discovered by the method used) found in the experimental results? That would be interesting to discuss.

Is the work clearly and accurately presented and does it cite the current literature?

Partly

If applicable, is the statistical analysis and its interpretation appropriate?

Yes

Are all the source data underlying the results available to ensure full reproducibility?

Yes

Is the study design appropriate and is the work technically sound?

Yes

Are the conclusions drawn adequately supported by the results?

Yes

Are sufficient details of methods and analysis provided to allow replication by others?

Yes

Reviewer Expertise:

Text and data mining

I confirm that I have read this submission and believe that I have an appropriate level of expertise to confirm that it is of an acceptable scientific standard.
